# Nujiangexanthone A Inhibits Hepatocellular Carcinoma Metastasis via Down Regulation of Cofilin 1

**DOI:** 10.3389/fcell.2021.644716

**Published:** 2021-03-12

**Authors:** Li Zhang, Zongtao Chai, Siyuan Kong, Jiling Feng, Man Wu, Jiaqi Tan, Man Yuan, Gan Chen, Zhuo Li, Hua Zhou, Shuqun Cheng, Hongxi Xu

**Affiliations:** ^1^School of Pharmacy, Shanghai University of Traditional Chinese Medicine, Shanghai, China; ^2^Department of Hepatic Surgery VI, Eastern Hepatobiliary Surgery Hospital, Second Military Medical University, Shanghai, China; ^3^Shuguang Hospital, Shanghai University of Traditional Chinese Medicine, Shanghai, China

**Keywords:** hepatocellular carcinoma, cofilin 1, cancer metastasis, Nujiangexanthone A, actin

## Abstract

Hepatocellular carcinoma (HCC) is one of the malignant tumors with poor prognosis. High expression level of cofilin 1 (CFL1) has been found in many types of cancers. However, the role of CFL1 in HCC hasn’t been known clearly. Here, we found that CFL1 was up regulated in human HCC and significantly associated with both overall survival and disease-free survival in HCC patients. Nujiangexanthone A (NJXA), the caged xanthones, isolated from gamboge plants decreased the expression of CFL1, which also inhibited the migration, invasion and metastasis of HCC cells *in vitro* and *in vivo.* Down regulation of CFL1 inhibited aggressiveness of HCC cells, which mimicked the effect of NJXA. Mechanism study indicated that, knockdown of CFL1 or treatment with NJXA increased the level of F-actin and disturbed the balance between F-actin and G-actin. In conclusion, our findings reveal the role of CFL1 in HCC metastasis through the CFL1/F-actin axis, and suggest that CFL1 may be a potential prognostic marker and a new therapeutic target. NJXA can effectively inhibit the metastasis of HCC cells by down regulating the expression of CFL1, which indicates the potential of NJXA for preventing metastasis in HCC.

## Introduction

In recent years, with the increasing incidence rate and mortality rate of many malignant tumors, cancer has become one of the most important causes of human death worldwide. Hepatocellular carcinoma (HCC) is one of the most common malignant tumors in the liver. Global cancer statistics show that the incidence rate and mortality rate of HCC ranks sixth and fourth, respectively, in malignant tumors (Bray et al., 2018). According to World Health Organization (WHO), more than one million patients will die from HCC worldwide by 2030. The cases in China account for more than 50% of all global liver cancer cases (Valery et al., 2018). At present, surgery is still the most effective treatment for HCC (Chen and Huang, 2005). However, the indications and role of surgery in the management of HCC are limited or with poor prognosis, since the symptoms of HCC patients at early stage are not typical, and patients are always in the middle or late stage when they are diagnosed (Kanda et al., 2015). The 5 year recurrence rate of HCC patients is about 70% (Llovet et al., 2018), and the 5 year survival rate is only 15∼34% (Yang et al., 2018).

Cofilin1 (CFL1) is a low molecular weight (21 KD) actin binding protein, which is widely found in eukaryotes. CFL1 is an important member of the actin depolymerizing factor (ADF) family, which plays an important regulatory role in the depolymerization and polymerization of actin and the remodeling of cytoskeleton (Gressin et al., 2015; Wang et al., 2015). Some studies have shown that the activation of CFL1 is closely related to the malignant invasive properties of tumor cells (Tahtamouni et al., 2013; Ling et al., 2020; Zhang et al., 2020; Wang J. et al., 2021). Inhibition of CFL1 activity can reduce the movement and migration of tumor cells, which may be an important target for inhibiting tumor growth and proliferation (Croise et al., 2014). Recently, much attention has been paid to the expression and regulation of CFL1 in malignant tumors. Existing research shows that CFL1 plays a very important role in the occurrence, development, prognosis, metastasis, epithelial mesenchymal transformation process, and drug resistance (Wang et al., 2006).

Studies indicated that the extracts and compounds from Traditional Chinese medicines suppressed the EMT (Zhao and Wei, 2015), proliferation and migration of different cancer cells by inhibiting CFL1 signaling pathway in different cancer cells (Wang et al., 2017; Liu et al., 2018). The *Garcinia* species from *Guttiferae* family have been studied for nearly 80 years (Lu et al., 2020; Wu et al., 2020). The major compounds of these species were xanthones, polycyclic polyprenylated acylphloroglucinols (PPAPs) and benzophenones (Han and Xu, 2009), which show a variety of biological functions (Wang et al., 2008; Xia et al., 2012). The novel compound Nujiangexanthone A (NJXA) was isolated from the leaves of *Garcinia nujiangensis*, which is a Chinese endemic species mainly distributed in the southwest region of China. Our previous studies have shown that NJXA is cytotoxic to a variety of human tumor cell lines, especially in cervical cancer. The compound inhibited cervical cancer cell growth by suppressing hnRNPK expression, which activated the c-Myc-cyclin/Cdk-Rb-E2F1 pathway and then caused cell cycle arrest (Zhang et al., 2016a).

## Materials and Methods

### Patients and Tissue Samples

HCC tissues and paracancerous tissues were collected from patients and the samples were histopathologically and clinically diagnosed. All patients underwent surgical resection at the Eastern Hepatobiliary Surgery Hospital between 2012 and 2017 and did not receive any anti-cancer therapy before surgery. The study protocol was approved by the Clinical Research Ethics Committee of Eastern Hepatobiliary Surgery Hospital, and all patients signed a written informed consent. After staining, the patients were divided into high CFL1 expression group and low CFL1 expression group according to the median (62:62). LIHC mRNA expression data was downloaded from the Cancer Genome Atlas (TCGA) data portal, and HCC patients were screened for this study^1^.

### Immunohistochemical Assay

According to our previous study (Chai et al., 2015), immunohistochemistry staining of paraffin-embedded tumor tissues was performed on the sections or tissue arrays containing tumor tissues from patients with HCC who had antibodies against CFL1 (1:100; Abcam# ab124979). The total area of positive staining was quantified by Image-Pro Plus software. For the reading of each antibody staining, a uniform setting for all the slides was applied.

### Cell Culture

Human hepatic cancer carcinoma Hep3B and HepG2 cell lines were obtained from the Chinese Academy of Sciences Committee Type Culture Collection Cell Bank. Cells were maintained in Dulbecco’s modified Eagle’s medium (Gibco/Invitrogen, 12800-017), supplemented with 10% fetal bovine serum (FBS) (Biological Industries, 04-001-1ACS, Beit HaEmek, Israel) and 10 U/ml penicillin-streptomycin (Gibco/Invitrogen, 15140-122). Cells were maintained in a standard humidified atmosphere of 5% CO_2_ at 37°C.

### Wound Healing Assay

Cells were seeded into a 24-well culture plate with the number of 1 × 10^5^ cells/well. When the cell density reached 90% confluence, a cell monolayer was scratched gently with a sterile pipette tip. After washing with PBS, fresh medium with or without NJXA was added. Cell migration was observed under IX83 microscope and imaging was performed at different time points (Olympus, Tokyo, Japan).

### Transwell Migration Assay

Transwell chambers (Corning, NY, United States) with an aperture of 8 μm were used to estimate cell migration. A total of 5 × 10^4^ cells were suspended in 100 μL serum-free medium and then added into the upper chamber, whereas 600 μL of complete medium was added to the lower chamber. After incubation with different concentrations of NJXA for 48 h, the cells in the upper cavity were removed with cotton swabs, and the migrated cells were fixed with methanol and stained with 0.1% crystal violet. Five fields were randomly selected and the number of migrated cells was counted under a microscope.

### Matrigel Invasion Assay

As for the transwell invasion assay, the upper chamber membranes were coated with matrigel according to the manufacturer’s instructions (BD Biosciences, Bedford, MA, United States). 1 × 10^5^ cells were added into the upper chamber in serum-free medium, the rest was the same as the migration assay.

### siRNA and Plasmid Transfection

CFL1-specific siRNAs (siRNA 260: 5′-GGUGUCAUCAAGGUG UUCATT-3′, 5′-UGAACACCUUGAUGACACCTT-3′; siRNA 438: 5′-CCACCUUUGU CAAGAUGCUTT-3′, 5′-AGCAUCU UGACAAAGGUGGTT-3′; siRNA 481: 5′-CCUCUAUGAUGC AACCUAUTT-3′, 5′-AUAGGUUGCAUCAUAGAGGTT -3′) and negative control siRNA (5′-UUCUCCGAACGUGUCA CGUTT-3′, 5′-ACGUG ACACGUUCGGAGAATT-3′) were purchased from GenePharma (Shanghai, China). CFL1 plasmid (GC362) was purchased from GenePharma (Shanghai, China). The transfection of siRNAs and plasmid was performed using Lipofectamine 2000 (Invitrogen) according to the manufacturer’s protocol.

### Western Blot Analysis

The cell lysate was collected and 20 μg of protein was subjected to SDS–PAGE, and then proteins were transferred to a polyvinylidene difluoride (PVDF) membrane (Millipore, Billerica, MA, United States). TBS/T (0.1%) containing 5% non–fat milk was used to block non–specific binding, the membrane was incubated in different primary antibodies: CFL1 (CST #5175), F–actin (Abcam#ab130935), G–actin (Abcam#ab123034), GAPDH (Abcam#ab128915). All antibodies were used at a 1:1,000 dilution, except for GAPDH, which was used at a 1:10,000 dilution. The membrane was washed with TBS/T four times to remove the unbound antibody and then incubated with the HRP–conjugated secondary antibodies at room temperature for 1 h. Protein bands were visualized with an ECL kit (Pierce, Rockford, IL, United States).

### RNA Extraction and Quantitative RT–PCR

Total RNA was isolated using Trizol reagent (Beyond, R0016) according to the manufacturer’s protocol. Reverse transcription PCR was carried out using PrimeScript RT Reagent Kit (Takara Biotechnology, China). Quantitative PCR analysis was conducted using SYBR Green real–time PCR kit (TOYOBO, QPK–201) in a Veriti Thermal Cycler (Applied Biosystems, Life Technologies). A StepOnePlus Real–Time PCR System Thermal Cycling Block was used for data collection (Applied Biosystems, Life Technologies). The primers for the qPCR reactions were as follows: CFL1, 5′ TGCTGCCAGATAAGGACTGC 3′ and 5′ CTCTTAAGGGGCGCAGACTC 3′; 18S, 5′-GTAACCC GTT GAACCCCATT-3′ and 5′-CCATCCAATCGGTAGTAGCG-3′. The PCR reaction conditions were 10 s at 95°C followed by 40 cycles of 5 s at 95°C and 20 s at 60°C.

### *In vivo* Animal Study

The use of all animals in our experiment was approved by the Institutional Animal Care and Use Committee of Shanghai University of Traditional Chinese Medicine. The sample size is estimated based on the sum of the minimum sample sizes required for each experiment. HepG2 cells (1 × 10^6^ cells per mouse) were injected intravenously into the 6 week-old BALB/c male nude mice (Experimental Animal Center of Chinese Academy of Sciences, Shanghai, China). After injection, the mice were randomly divided into three groups (n = 6 in each group) and received an intraperitoneal injection of vehicle control (1% Tween-80 in saline), NJXA (20 mg/kg) or 5-FU as the positive control (20 mg/kg). The vehicle and NJXA were injected every day, while 5-FU was treated every 2 days. The body weight of the mice was measured every other day. At day 25, the mice were sacrificed, and the lungs were removed and fixed in Bouin’s solution. The number of pulmonary nodules was blindly counted and confirmed by HE staining. Immunohistochemical staining of CFL1, F-actin and G-actin were performed in the lungs. Each section was examined independently by two investigators in a blinded manner.

### Statistical Analyses

All experiments in this study were performed in triplicate and repeated at least three times, data were presented as the means ± SD from three independent experiments. The data satisfied the normality and statistical analysis was performed using a 2-tailed Student’s *t-*test. Univariate Cox analysis was first carried out with independent variables followed by a multivariate Cox analysis of the variables with *P* < 0.05 in univariate Cox analysis. The multivariable Cox model was built using SPSS with backward conditional selection *P*-values less than 0.05 were considered to be statistically significant.

## Results

### The Expression of CFL1 Correlates With Tumor Metastasis in HCC

**FIGURE 1:**
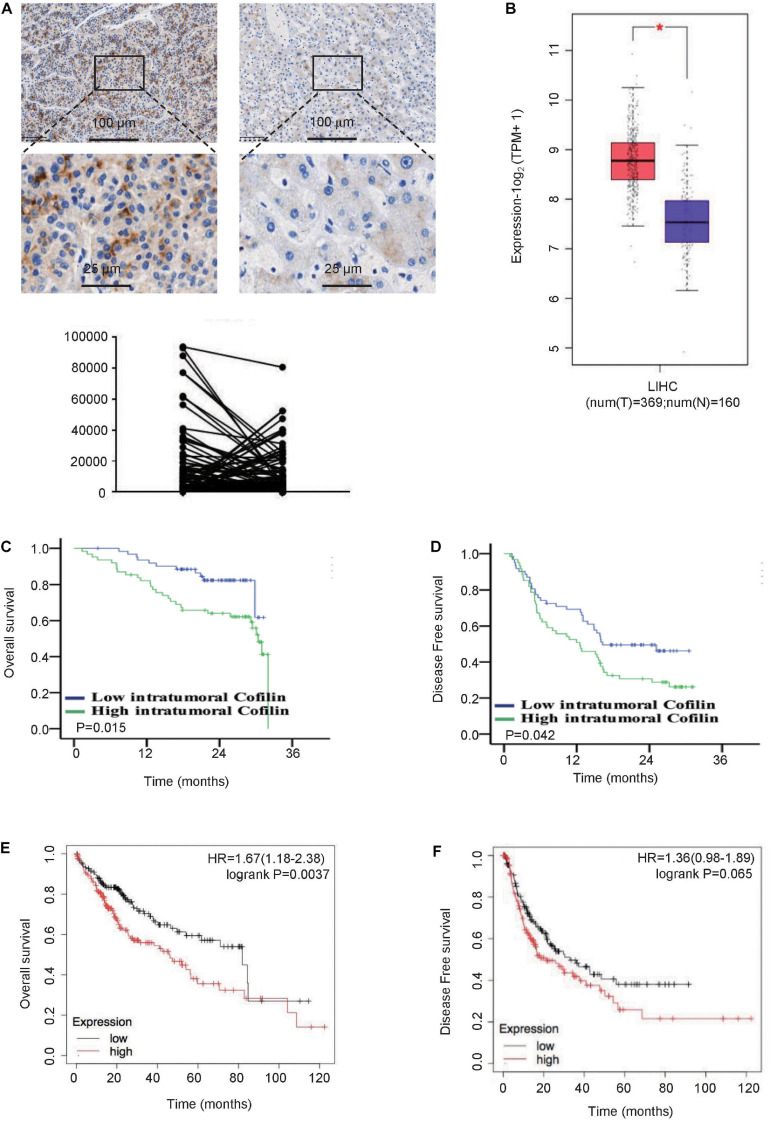
High expression of CFL1 is associated with poor survival in HCC patients. **(A)** Protein levels of CFL1 in 124 paired HCC and adjacent tissues were detected by immunohistochemistry. Paired samples *t*-test was used. **(B)** The mRNA expression level of CFL1 in TCGA and GTEx’s liver hepatocellular carcinoma (LIHC) dataset including 160 tumor free tissues and 369 tumor tissues. The box plot is generated by GEPIA2 with jitter (size = 0.4). Red cluster: tumor samples; blue cluster: normal samples. Genes with higher | log2FC| values (>1) and lower *Q*–values (<0.01) were considered differentially expressed genes and marked the symbol ^∗^. Mean OS **(C)** and DFS **(D)** between patients with high and low CFL1 expression in our cohort. Mean OS **(E)** and DFS **(F)** between patients with high and low CFL1 expression from TCGA. Kaplan Meier analysis and log rank test were used.

The clinicopathological characteristics of the 124 patients are summarized in Table 1. We first compared the expression level of CFL1 between tumor tissues and paracancerous tissues. We found that the protein level of CFL1 in tumor tissues was higher as compared to tumor free tissues, however, there is no significant difference (Figure 1A). Since sample amount was limited, we further determined the CFL1 expression level in human HCC using TCGA datasets. The results showed that the mRNA level of CFL1 was significantly increased in tumor tissues as compared with tumor free tissues (Figure 1B). In order to better understand the relationship between CFL1 and HCC, the patients were divided into two groups, the CFL1 low-expression group (*n* = 62) and high-expression group (*n* = 62). The results showed that the number of tumor (*P* = 0.040), portal vein tumor thrombus (PVTT) (*P* = 0.005), and microvascular invasion (MVI) (*P* = 0.009) were closely related with CFL1 expression (Table 1). MVI is a powerful, validated, independent predictor of early recurrence and poor overall survival (OS) after surgical treatment for HCC (Roayaie et al., 2009; Lim et al., 2011). PVTT is an independent predictor of distant metastasis and promotes distant metastasis of HCC (Bruix and Sherman, 2005, 2011; Addario et al., 2011), which increases the mortality of patients with HCC and PVTT. These results suggested that high expression of CFL1 might promote the migration and invasion of HCC cells, and may be associated with poor prognosis.

**TABLE 1:** Relationships between intratumoral CFL1 expression and clinicopathological variables.

Clinical variables	High expression (*n* = 62)	Low expression (*n* = 62)	*P*
Age, year	50.05 ± 9.16	47.83 ± 11.26	0.265
Sex			0.084
Male	55	60	
Female	7	2	
HBsAg			0.729
No	4	5	
Yes	58	57	
Tumor diameter	6.540 ± 3.65	6.410 ± 3.20	0.833
No of tumor			0.040
Single	52	59	
Multiple	10	3	
PVTT			0.005
No	50	60	
Yes	12	2	
Encapsulation			0.857
No	27	28	
Yes	35	34	
Liver Cirrhosis			0.379
No	8	5	
Yes	54	57	
Ascites			0.752
No	57	56	
Yes	5	6	
Microvascular invasion			0.009
No	32	46	
Yes	30	16	
TBIL	14.79 ± 6.46	13.67 ± 4.69	0.272
DBIL	5.71 ± 2.70	5.42 ± 1.75	0.479
ALB	42.80 ± 5.87	43.11 ± 3.61	0.727
ALT	44.40 ± 24.07	47.40 ± 27.58	0.520
PT	12.10 ± 1.06	11.94 ± 1.12	0.412
GGT	107.19 ± 117.42	92.56 ± 69.57	0.400
ALP	113.79 ± 91.89	95.61 ± 42.15	0.159
AFP	616.85 ± 427.73	555.54 ± 523.66	0.053
CA199	25.86 ± 27.38	24.79 ± 22.71	0.813
CEA	2.80 ± 2.80	105.49 ± 798.98	0.316
AST	46.76 ± 30.85	41.92 ± 25.77	0.345

*HBsAg, Hepatitis B surface antigen; AFP, α-fetoprotein; TBIL, Total Bilirubin; DBIL, Direct Bilirubin; ALB, Albumin; ALT, Alanine Aminotransferase; AST, Aspartate Aminotransferase; GGT, γ-Glutamyltransferase; ALP, Alkaline phosphatase; PT, Prothrombin time; CEA, Carcinoembryonic antigen; CA199, Carbohydrate Antigen 19-9.*

### High Expression of CFL1 in Tumor Tissues Is Related With Poor Survival in Patients

To evaluate the relationship between CFL1 expression in tumor tissues and prognosis of HCC patients, Kaplan Meier survival analysis was used. In patients with high expression of CFL1, 29 out of 62 patients died and 44 experienced recurrence, while only 23 out of 62 patients died and 32 recurrences in patients with low CFL1 expression (Figures 1C,D). For the HCC patients from TCGA, log rank analysis also demonstrated that HCC patients with high CFL1 expression had poorer overall survival, which was 45.7 and 81.9 months (*P* = 0.0037; Figure 1E) in patients with high and low CFL1, respectively, and the recurrence-free survival was 21.23 and 33 months (*P* = 0.065; Figure 1F), these results also verified our findings.

In order to further investigate whether CFL1 is an independent risk factor for OS and DFS, the univariate and multivariate analyses were made using the Cox proportional hazards model (Table 2). The univariate analysis indicated that higher expression of CFL1 led to poorer OS (*p* = 0.015) and DFS (*p* = 0.042) while the multivariate analysis results showed that, CFL1 was an independent risk factor for OS (*p* = 0.002) but not DFS. In conclusion, our study showed that high expression of CFL1 was closely related to malignant progression and poor clinical outcomes.

**TABLE 2:** Multivariate analysis of factors associated with survival and recurrence.

	OS				DFS			
	Multivariate				Multivariate			
Features	Univariate *P*	HR	95% CI	*P*	Univariate *P*	HR	95% CI	*P*
α-fetoprotein: > 200 ng/dL vs. ≤ 200 ng/dL	0.026	0.430	0.164–1.126	0.086	0.033			NS
No of tumor	0.000	0.108	0.020–0.580	0.010	0.005	0.210	0.024–1.818	0.157
Tumor size: >5 cm vs. ≤5 cm	0.004	0.230	0.072–0.734	0.013	0.003			NS
Encapsulation: complete vs. none	0.004	0.314	0.114–0.863	0.025	0.002			NS
Microvascular invasion: yes vs. no	0.000			NS	0.000	0.321	0.133–0.777	0.012
PVTT	0.005			NS	0.002	0.169	0.020–1.427	0.102
Ascites	0.001	0.209	0.041–1.066	0.060				
Intratumoral cofilin high vs. low	0.015	0.198	0.072–0.847	0.002	0.042			NS

*CI, confidence interval; HR, hazard ratio.*

### Down-Regulation of CFL1 Inhibits HCC Cells Migration and Invasion

**FIGURE 2:**
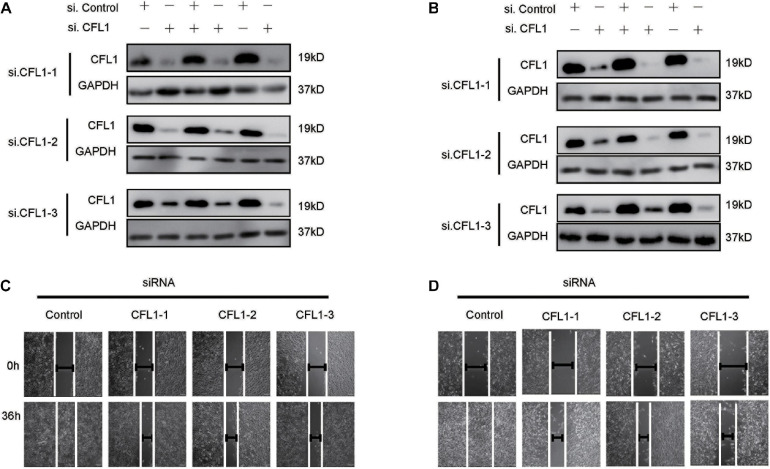
Silencing CFL1 inhibited migration and invasion of HCC cells. **(A)** HepG2 or **(B)** Hep3B cells were transfected with individual CFL1-specific siRNAs or control siRNA for 24–72 h, and cell lysates were collected and subjected to western blotting. After transfection, a scratch was made in monolayers of **(C)** HepG2 or **(D)** Hep3B cells, and the migration ability of the cells was detected with an inverted microscope.

To further study the role of CFL1 in the metastasis of HCC cells, we conducted loss-of-function studies. First, the protein of CFL1 was silenced to investigate whether the knockdown of CFL1 could inhibit migration or invasion of HCC cells. HepG2 and Hep3B cells were transiently transfected with specific siRNAs. As shown in Figures 2A,B, after transfection the expression of CFL1 was greatly down regulated in both cell lines (Figures 2A,B). We then used the transfected cells to further evaluate the effect of CFL1 on cell mobility. In the wound healing assay, knockdown of CFL1 reduced cell migration by about 70% over 48 h as compared to cells transfected with control siRNA (Figures 2C,D). Our later study showed that similar results were observed for transwell and matrigel assays in both HepG2 and Hep3B cells.

### NJXA Decreased the Expression of CFL1 in HCC Cells

**FIGURE 3:**
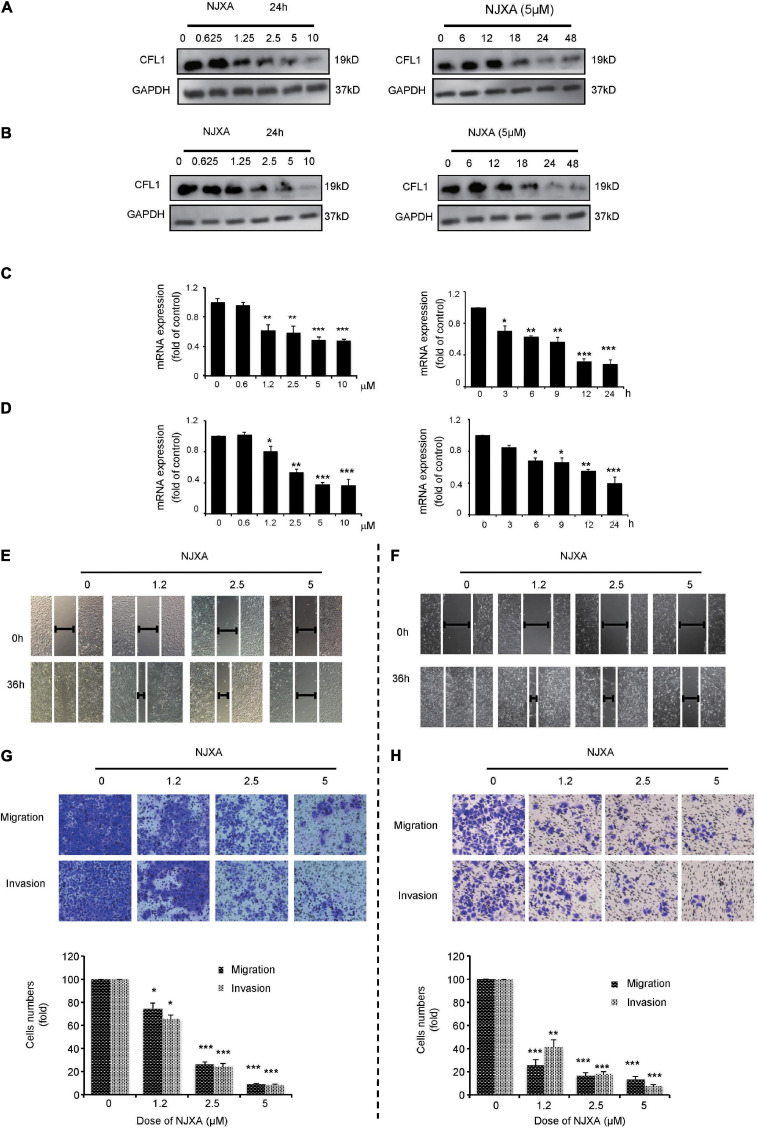
NJXA inhibited expression of CFL1 and metastasis in HCC cells. **(A)** HepG2 or **(B)** Hep3B cells were administrated with NJXA for indicated time. Cells lysates were collected and analyzed by Western blotting for CFL1 and GAPDH. The relative CFL1 mRNA levels in **(C)** HepG2 or **(D)** Hep3B were analyzed by qRT-PCR after treatment of NJXA; and the relative CFL1 mRNA levels (normalized to 18S rRNA levels) were determined. Wound healing assay. **(E)** HepG2 or **(F)** Hep3B cells were scratched and treated with NJXA. Images were observed by an inverted microscope. The migration and invasion ability of **(G)** HepG2 or **(H)** Hep3B was detected by transwell or matrigel invasion assay cells after treatment of NJXA. Cells were fixed and stained with 0.1% crystal violet. The cell number was counted for each group in three independent experiments. All the data are presented as means ± SD. ***P < 0.001 compared to the control (*n* = 3).

Our previous studies have shown that caged xanthone NJXA, isolated from *Garcinia nujiangensis*, showed antitumor activities on several cell lines. Proteomics study indicated that CFL1 was one of the several proteins that were significantly suppressed by NJXA in HeLa cells. Here, in our study we first investigated whether NJXA could also down regulate the expression of CFL1 in HCC cells. The protein and mRNA levels of CFL1 in HCC cells were detected by Western blotting and real-time PCR. Results showed that treatment of NJXA decreased both the protein (Figures 3A,B) and mRNA level (Figures 3C,D) of CFL1 in a dose and time-dependent manner in HepG2 and Hep3B cell lines. The phosphorylated level of CFL1 was also determined in our study, results showed that NJXA could also decrease the phosphorylation of CFL1 in HCC cells (Supplementary Figure 1).

### NJXA Inhibited the Metastasis in hepG2 and hep3B Cells

Based on our results, the expression of CFL1 was down regulated and this correlates with tumor metastasis in HCC patients. Herein we investigated whether NJXA could inhibit cell migration and invasion in HCC cells. In order to know whether the suppression of tumor metastasis was caused by cell proliferation inhibition or cell cycle arrest, the effect of NJXA on HCC cells was first detected by MTT assay and flow cytometry test. As shown in Supplementary Figures 2, 3, 5 μM of NJXA did not inhibit cell growth within 48 h, neither did NJXA show effect on cell cycle distribution on both cell lines. Therefore, we chose 5 μM as the maximum dosage for the following mechanism study. Results showed that exposure to NJXA decreased the numbers of migrated HCC cells in a dose-dependent manner (Figures 3E,F). In order to further confirm the effect of NJXA on cell migration, we tested whether NJXA could inhibit cell migration in Transwell experiment. As shown in Figures 3G,H, NJXA dose-dependently reduced the number of migrated cells. In addition, the Transwell invasion assay with matrix gel showed that the number of invasive cells after NJXA treatment was greatly decreased. These results indicate that NJXA can inhibit the metastasis of HepG2 and Hep3B cells *in vitro*, but has no obvious cytotoxicity.

### NJXA Inhibited Cell Metastasis Partially Through Down-Regulation of CFL1 Protein

**FIGURE 4:**
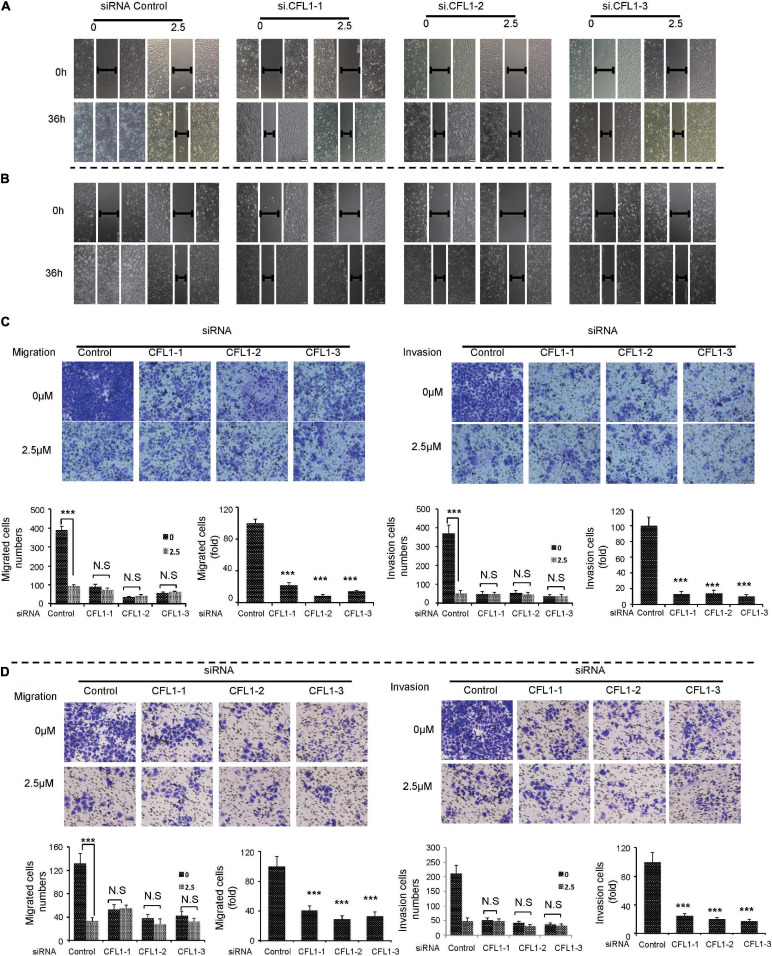
NJXA inhibited cell metastasis partially through down-regulation of CFL1 protein. After transfection, HepG2 or Hep3B cells were treated with or without NJXA (2.5 μM) for 36 h. Cell migration ability of **(A)** HepG2 or **(B)** Hep3B was examined by wound healing assay, cell migration or invasion of **(C)** HepG2 or **(D)** Hep3B was measured by transwell or matrigel coated transwell assays. The migrating or invading cells were fixed and stained with 0.1% crystal violet. The numbers of cells in migration and invasion assays were counted for each group in three independent experiments. Data are shown as the means ± SD. ^∗^*P* < 0.05, ^∗∗^*P* < 0.01, and ^∗∗∗^*P* < 0.01 compared to the control (*n* = 3).

Since NJXA greatly decreased HCC metastasis as well as the expression of CFL1, we supposed that CFL1 was partially involved in this inhibitory effect. In order to verify our hypothesis, we applied NJXA to CFL1 knocked down cells. In consistent with our results above, silencing the protein of CFL1 could significantly decrease HCC cells migration and invasion (Figure 4). Moreover, the inhibitory effects of NJXA on control cells and CFL1 knockdown cells were compared by wound healing assay, transwell migration assay and transwell invasion assay. The results showed that the effect of NJXA on cell metastasis was greatly decreased in CFL1 knocked down cells as compared with cells transfected with control siRNA (Figure 4). Next, we want to know whether overexpression of CFL1 could attenuate the inhibitory effect of NJXA. However, our results showed that the cell migration was decreased after overexpression of CFL1 (Supplementary Figure 4), which was consistent with previous study that overexpression of CFL1 suppressed growth and invasion of cancer cells (Tsai et al., 2015).

### CFL1-Mediated Dynamics of Actin Cytoskeleton Is Involved in NJXA-Regulated Migration and EMT in HCC Cells

**FIGURE 5:**
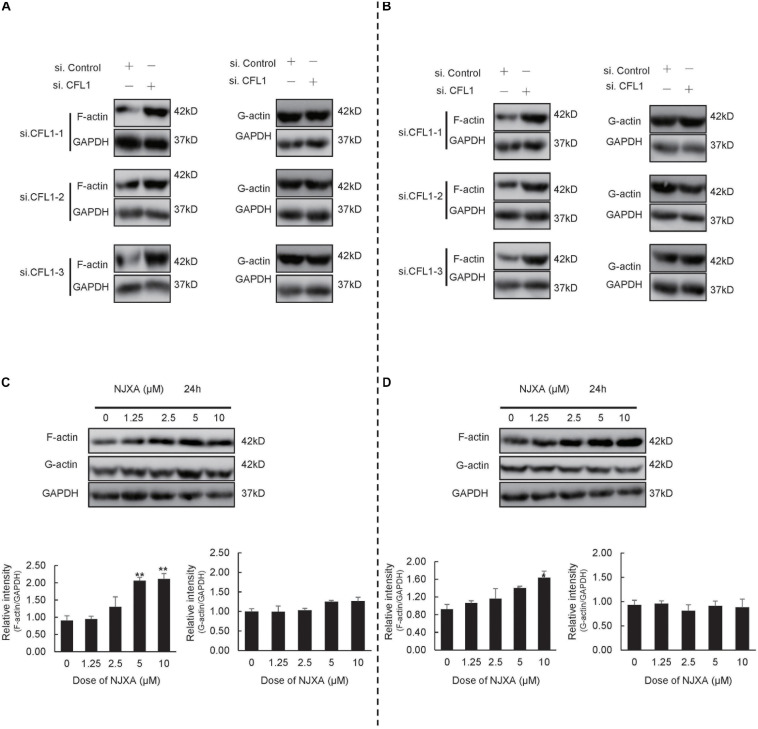
NJXA inhibited cell metastasis partially by disturbing the balance between G-actin and F-actin. **(A)** HepG2 or **(B)** Hep3B cells were transfected with control siRNA or CFL1-specific siRNAs for 48 h, and cell lysates were analyzed by western blotting for protein expression of G-actin, F-actin and GAPDH. **(C)** HepG2 or **(D)** Hep3B cells were treated with different concentrations of NJXA for 24 h, the protein expression of G-actin, F-actin and GAPDH was analyzed by western blotting. All the data are presented as means ± SD. *P < 0.05 and **P < 0.01 compared to the control (*n* = 3).

CFL1, the actin-binding protein, maintains the dynamic balance between depolymerization and polymerization of actin. To further study the mechanisms by which NJXA and CFL1 inhibit cells metastasis, we determined the expression of F-actin and G-actin after NJXA treatment. As shown in Figures 5A,B, after silencing of CFL1, the expression of F-actin was increased, while no obvious change was found in G-actin. Similar results were observed in HCC cells after treatment of NJXA (Figures 5C,D). The protein levels of actin-binding protein complex Arp 2/3, a key actin filament nucleation factor, were also detected. However, NJXA showed no effect on these two proteins (Supplementary Figure 5), which indicated that NJXA showed no obvious effect on actin nucleation. Therefore, the decrease in CFL1 expression after treatment of NJXA mainly destroyed the balance between F-actin and G-actin, which then further inhibited the cancer metastasis. Actin cytoskeleton not only plays an important role in cell migration, which has also been reported to regulate EMT in metastatic cancer (Shankar and Nabi, 2015; Li et al., 2020). Our study here also found that after treatment with NJXA, the expression of EMT markers was down regulated, such as snail and vimentin, while E-cadherin was up regulated (Supplementary Figure 6). These results suggested that NJXA might regulate HCC metastasis through CFL1 mediated dynamics of actin cytoskeleton.

### NJXA Suppressed Pulmonary Tumor Metastasis in Nude Mice

**FIGURE 6:**
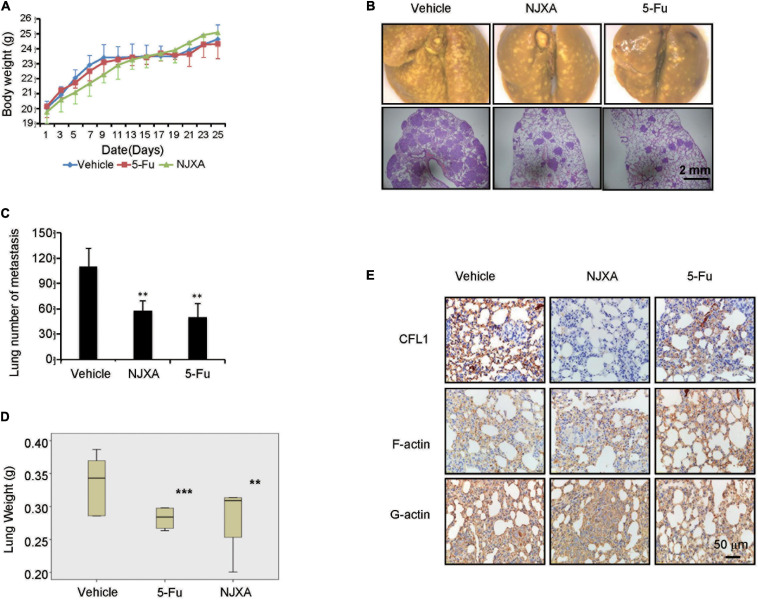
NJXA suppressed pulmonary tumor metastasis in mice. Six week-old male nude mice were injected with 1 × 10^6^ HepG2 cells *via* tail veins. After injection the mice were randomly divided into three groups (*n* = 8), and treated with vehicle control (1% DMSO in normal saline), NJXA (20 mg/kg), or 5-FU (2 mg/kg every 2 days) via intraperitoneal injection. After treatment for 16 days, the mice were sacrificed. **(A)** Body weight analysis every 2 days during the whole experiments. **(B)** Representative lung images and HE staining of lungs from each group. **(C)** Quantitative analysis of metastatic nodes in lung. **(D)** Lung weight analysis after treatment. **(E)** Immunohistochemistry staining of CFL1, F-actin and G-actin in lung tissues. All the data are presented as means ± SD. **P < 0.01 and ***P < 0.001 compared to the control (n = 3).

In order to determine the anti-metastatic effect of NJXA *in vivo*, pulmonary metastasis mouse model was used by tail vein injection of HepG2 cells in nude mice. After injection of HepG2 cells, NJXA (20 mg/kg, i.p.) was administered daily for 3 weeks, while 5-FU (20 mg/kg, i.p.) was given every 2 days. After treatment for 3 weeks, mice were sacrificed, the side effect and anti-metastasis effect of NJXA were investigated. During the treatment, there was no significant change in body weight between vehicle-treated and the NJXA-treated groups (Figure 6A). NJXA didn’t induce apparent cellular change or hemorrhage in the heart, lung, kidney or liver (Supplementary Figure 7). Tumor nodules and HE staining of lung tissues were used to evaluate the effect of NJXA on pulmonary metastasis. As shown in Figure 6B (upper panel), tumor nodules were observed in all the three groups. However, the number was greatly decreased after the treatment of NJXA or 5-FU, and statistical results were showed in Figure 6C. In consistent with the result of tumor nodules, the HE staining results demonstrated that the size of metastasis was much larger in vehicle control group as compared with NJXA or 5-FU treated groups, illustrated in Figure 6B (lower panel). In addition, NJXA also exhibited inhibitory effect on lung weight. The mean lung weight measured on day 24 for the vehicle control, NJXA and 5-FU groups were 0.369, 0.283, and 0.282, respectively (Figure 6D). The results indicated that the anti-metastatic effect of NJXA on tumor was similar to that of 5-FU.

In order to illustrate the molecular mechanism of NJXA on tumor metastasis, the protein expression levels of CFL1, F-actin and G-actin were examined in tumor tissues by immunohistochemical analysis. As shown in Figure 6E, the expression of CFL1 in the lung tissue of NJXA treated mice was decreased as compared with the control group, while the proteins of F-actin and G-actin did not show obvious change after the treatment of NJXA. In conclusion, our studies showed that NJXA inhibited pulmonary metastasis in nude mice without obvious side effects on major organs.

## Discussion

In this work, we found that up-regulation of CFL1 was significantly associated with shorter overall survival and disease-free survival of HCC patients. Knockdown of CFL1 inhibited cell invasion and migration of hepatocarcinoma cells. The natural compound NJXA inhibited HCC cells migration and invasion *in vitro* and metastasis *in vivo* by down regulation of CFL1. Our results suggest that CFL1 may be a promising prognostic biomarker and new therapeutic target for HCC, and the natural xanthone NJXA might be a potential inhibitor of CFL1 in HCC.

Cell migration is important for tumor invasion and metastasis (Croise et al., 2014). In recent years, many studies have found that CFL1 plays an important role in movement of tumor cells, the regulation of adhesion between tumor cells and extracellular matrix, the mitosis of tumor cells, and the speed and depth of tumor cell invasion (Zhang et al., 2015). It is found that CFL1 is closely related to occurrence and development of certain cancers, which may be a new biomarker and treatment target for early diagnosis and prognosis. For example, studies showed that the pathological classification and clinical stage of esophageal cancer was closely related to the expression of CFL1 (Wang et al., 2010). It was also reported that CFL1 was closely related to the size, stage and differentiation of gastric cancer, especially to the invasion, and prognosis of gastric cancer (Yan et al., 2010; Cho et al., 2012; Wu et al., 2012). Herein, we first found that CFL1 significantly correlated with the markers that were closely related to HCC tumor metastasis, showing that CFL1 may also play an important role on metastasis in HCC. Therefore, the key role of CFL1 in HCC cells was investigated. Knockdown of CFL1 significantly inhibited the invasion and migration of HCC cells, which was consistent with clinical features. In addition, Kaplan-Meier survival curve and log rank test showed that the overall survival rate of patients with high expression of CFL1 was lower, as well as the disease-free survival rate. Our results suggested that high expression of CFL1 might promote the occurrence and metastasis of HCC.

Cell cytoskeleton plays an important role in maintaining the shape of cells, as well as in cell movement. Microfilament is one of the main components of cytoskeleton, which is mainly composed of actin that exists in the form of monomer (G-actin) and polymer (F-actin). CFL1 is essential for severing actin filaments, which increase the pool of G-actin, as well as to form free barbed ends of actin filaments for polymerization (Lappalainen and Drubin, 1997; Bamburg et al., 1999; McGrath et al., 2000; Condeelis, 2001; Zhu et al., 2006). Therefore, CFL1 is an important regulator of actin dynamics. CFL1 regulates actin polymerization and depolymerization during cell movement, and destroying this balance can effectively inhibit cell migration. Previous studies showed that knockdown of CFL1 protein decreased the ability of cell motility, one of the reason was that CFL1 knockdown decreased the ratio of G-actin vs. F-actin(Hotulainen et al., 2005), and the G-actin pool may be diminished, affecting the fast recycling of actin filaments. In consistent with previous study, our results also showed that in HCC cells, silencing of CFL1 or incubation with NJXA increased the protein level of F-actin, and inhibited cell migration and invasion. Intuitively, an increase in CFL1 expression by transfection would expect to increase the metastasis of cancer cells. However, our study found that after over-expression of CFL1, the migration of cells was also inhibited. The reason might be that over-expression of CFL1 caused actin cytoskeletal destabilization, which then induced cell cycle arrest and cell proliferation reduction (Lee and Keng, 2005). Mechanistic study showed that suppression of cancer cell growth and invasion due to over-expression of CFL1 might be associated with up-regulation of let-7 microRNA in human non-small cell lung cancer (NSCLC) cells (Tsai et al., 2015).

The activity of CFL1 was mainly regulated by the phosphorylation and dephosphorylation at Ser-3 by different kinase. It is well known that the inhibition of LIM kinase 1 downregulated CFL1 phosphorylation, which then suppresses the motility of cancer cells (Lu et al., 2018). Recently, research found that the inactivation of the Src/Akt/mTOR pathway could also suppress the phosphorylation of CFL1, which then regulate the migration and EMT (Li et al., 2020; Kulkarni et al., 2021). It has been reported that induction of EMT promotes invasion and migration of cancer cells, and predicts poor prognosis in HCC patients (Wang G. et al., 2021). Our research here found that NJXA also inhibited the Src/Akt pathway by decreasing the phosphorylation of Src and Akt (Supplementary Figure 1). The expression of EMT markers was also regulated after treatment with NJXA, which decreased the protein levels of snail and vimentin while E-cadherin was up regulated. However, further study should be carried on to confirm whether the effect of NJXA on EMT markers was induced by inhibition of Src/Akt pathway.

NJXA is a novel compound from *Garcinia nujiangensis*. Our previous study showed that NJXA suppressed cervical cancer growth by inducing cell cycle arrest and cell apoptosis in cervical cancer (Zhang et al., 2016a, b). The major functions of NJXA may not be the same in different cell lines, as showed in our previous study, the IC_50_ of NJXA in cervical cancer cells was much lower than in HCC cells (Zhang et al., 2016a). Moreover, NJXA showed no effect on cell cycle distribution in HepG2 and Hep3B cells, all these results indicated that the major function of NJXA may not act on cell growth and cell cycle in HepG2 and Hep3B cells. Our study here proved that NJXA effectively inhibited cell migration and invasion *in vitro*, as well as strongly suppressed metastasis *in vivo* without apparent toxicity. Our previous proteomic study showed that CFL1 was one of the potential target proteins in HeLa cells and the mRNA as well as protein levels of CFL1 were significantly decreased after treatment of NJXA. Knockdown of CFL1 mimicked the inhibitory effect of NJXA on cell migration and invasion. Moreover, the effect of NJXA on cell mobility was greatly diminished in both HepG2 and Hep3B cells after transfected with CFL1-specific siRNAs, which further proved that NJXA inhibited cell migration and invasion mainly through the regulation of CFL1.

## Conclusion

In summary, our study demonstrated that high expression of CFL1 is closely related to tumor metastasis and poor clinical outcomes in human HCC. Knockdown of CFL1 in HCC cells greatly inhibited the cell mobility. NJXA, a natural xanthone, inhibited cell metastasis *in vitro* and *in vivo* partially through down regulation of CFL1. Our results demonstrated that CFL1 might be served as a new biomarker of HCC, and NJXA might provide a new therapeutic strategy in HCC.

## Data Availability Statement

Publicly available datasets were analyzed in this study. This data can be found here: https://portal.gdc.cancer.gov/genes/ENSG00000172757; https://portal.gdc.cancer.gov/projects/TCG A-LIHC.

## Ethics Statement

The studies involving human participants were reviewed and approved by the Clinical Research Ethics Committee of Eastern Hepatobiliary Surgery Hospital. The patients/participants provided their written informed consent to participate in this study. The animal study was reviewed and approved by the Institutional Animal Care and Use Committee of Shanghai University of Traditional Chinese Medicine. Written informed consent was obtained from the individual(s) for the publication of any potentially identifiable images or data included in this article.

## Author Contributions

HX, LZ, ZC, and SC conceived and designed the experiments. SK, JF, and MW performed the animal experiments. JT and MY did the *in vitro* experiments. GC and ZL analyzed the data. LZ, ZC, and HZ wrote the manuscript. All authors contributed to the article and approved the submitted version.

## Conflict of Interest

The authors declare that the research was conducted in the absence of any commercial or financial relationships that could be construed as a potential conflict of interest.

## Footnotes

1 https://tcgadata.nci.nih.gov/docs/publications/tcga/
